# The Influence of Lifestyle Modifications on Cardiovascular Outcomes in Older Adults: Findings from a Cross-Sectional Study

**DOI:** 10.3390/life15010087

**Published:** 2025-01-13

**Authors:** Mohammed Almutairi, Ashwaq A. Almutairi, Abdulaziz M. Alodhialah

**Affiliations:** 1 Department of Medical Surgical Nursing, College of Nursing, King Saud University, Riyadh 11451, Saudi Arabia; mohalmutairi@ksu.edu.sa; 2 School of Nursing & Midwifery, Monash University, Melbourne, VIC 3004, Australia; ashwaq.almutairi@monash.edu

**Keywords:** cardiovascular disease, diet, lifestyle modification, older adults, physical activity, smoking cessation

## Abstract

**Background:** Cardiovascular disease (CVD) is a leading cause of morbidity and mortality among older adults. Lifestyle modifications, including diet, physical activity, and smoking cessation, are key to reducing cardiovascular risk. This study examines the combined effects of these behaviors on cardiovascular outcomes and their mediating mechanisms. **Methods:** A cross-sectional study was conducted among older adults (aged ≥ 60 years) in Riyadh, Saudi Arabia. Data on dietary quality, physical activity, and smoking status were collected using validated questionnaires. Cardiovascular outcomes, including low-density lipoprotein cholesterol (LDL), systolic blood pressure (SBP), and body mass index (BMI), were measured. A composite cardiovascular risk score was computed. Path analysis was employed to assess direct and indirect effects of lifestyle factors on cardiovascular outcomes. **Results:** Participants adhering to a healthy diet, engaging in regular physical activity, and avoiding smoking had significantly lower composite cardiovascular risk scores. Non-smoking status showed the strongest direct effect (β = −0.20, *p* = 0.006), while dietary quality and physical activity exhibited significant indirect effects mediated by LDL, SBP, and BMI. Combined adherence to multiple healthy behaviors resulted in the greatest reductions in cardiovascular risk. The path analysis highlighted dietary quality and physical activity as critical mediators of cardiovascular health improvements. **Conclusions:** Lifestyle modifications significantly reduce cardiovascular risk in older adults, with cumulative benefits observed for combined adherence to healthy behaviors. These findings emphasize the importance of comprehensive lifestyle interventions targeting diet, physical activity, and smoking cessation to promote cardiovascular health in aging populations.

## 1. Introduction

Cardiovascular disease (CVD) remains a predominant cause of morbidity, mortality, and diminished quality of life worldwide, particularly among older adults who represent an ever-growing demographic segment in both developed and developing nations [1]. As global life expectancy continues to rise, individuals aged 65 years and above constitute a substantial proportion of the population, leading to an urgent need to understand and address the factors that influence their cardiovascular health outcomes [2,3]. Indeed, the increasing prevalence of CVD in later life stages places a significant burden not only on patients and their families but also on healthcare systems, necessitating more effective preventive measures and interventions that can be feasibly implemented in this older cohort [4].

The causes of CVD are multifactorial, involving a complex interplay between non-modifiable and modifiable risk factors. Non-modifiable risk factors include age, gender, and genetic predisposition. The risk of developing CVD increases with age, and while men are generally at higher risk at younger ages, the risk for women increases post-menopause [5]. Genetic factors can predispose individuals to hypertension, dyslipidemia, and other conditions that elevate CVD risk [6].

Modifiable risk factors are behaviors and conditions that individuals can change to reduce their CVD risk. These include hypertension, dyslipidemia, diabetes mellitus, obesity, smoking, physical inactivity, and unhealthy diet [7]. Hypertension, or high blood pressure, damages arterial walls and accelerates atherosclerosis, leading to increased risk of heart attack and stroke [8]. Dyslipidemia, characterized by elevated low-density lipoprotein (LDL) cholesterol and low high-density lipoprotein (HDL) cholesterol levels, contributes to plaque formation in arteries [9]. Diabetes mellitus exacerbates endothelial dysfunction and promotes inflammation, further increasing CVD risk [10].

Lifestyle factors play a pivotal role in the development and progression of CVD. Smoking introduces toxins that damage the cardiovascular system, leading to endothelial dysfunction and increased thrombosis [11]. Physical inactivity contributes to obesity, hypertension, and insulin resistance, all of which are risk factors for CVD [12]. An unhealthy diet rich in saturated fats, trans fats, sodium, and sugars can lead to hypertension, dyslipidemia, and obesity [13]. These factors not only increase the risk of developing CVD but also worsen outcomes in individuals with existing cardiovascular conditions.

The pathophysiology of CVD in older adults is complex, often involving the interplay of biological, behavioral, and environmental factors accumulated across the lifespan [14]. Traditional risk factors such as hypertension, hyperlipidemia, diabetes mellitus, and obesity remain central to the progression of CVD, and their prevalence tends to increase with advancing age [15]. However, beyond these well-established clinical determinants, lifestyle factors—most notably diet, physical activity, smoking habits, and alcohol consumption—have garnered increasing attention as modifiable risk factors capable of exerting a significant influence on cardiovascular outcomes [16]. Since the older population is frequently characterized by long-standing health behaviors, understanding how even late-life modifications in lifestyle can influence CVD risk has profound public health implications [17,18].

Lifestyle modification, broadly defined as a set of behavioral changes aimed at promoting better health, is considered a cornerstone of primary and secondary prevention strategies for CVD [19]. Numerous interventional and observational studies have demonstrated that adopting a balanced diet, engaging in regular physical activity, avoiding tobacco use, and limiting excessive alcohol intake can collectively reduce the risk of atherosclerosis, coronary artery disease, stroke, and heart failure [20,21]. For instance, adherence to dietary patterns such as the Mediterranean diet or the Dietary Approaches to Stop Hypertension (DASH) plan has been linked to better blood pressure control, improved lipid profiles, and reduced inflammation, all of which contribute to more favorable cardiovascular outcomes [22,23]. Similarly, a physically active lifestyle, whether through structured exercise programs or regular leisure-time activities such as walking, gardening, and cycling, has been consistently associated with improved cardiorespiratory fitness, better vascular endothelial function, and enhanced metabolic health, thereby mitigating CVD risk [24,25,26].

Smoking cessation at any age confers substantial benefits in terms of reducing cardiovascular mortality and morbidity, and these benefits extend into older adulthood [27,28]. Research shows that individuals who quit smoking, even in later stages of life, experience a significant reduction in the risk of myocardial infarction and stroke compared to those who continue to smoke, underscoring the immense value of cessation interventions [29]. Moderation of alcohol intake, too, has important implications. Although some evidence suggests light-to-moderate alcohol consumption may be cardioprotective, heavy alcohol use is clearly detrimental, exacerbating hypertension, arrhythmias, and cardiomyopathies [30]. Taken together, these lifestyle factors are modifiable targets, offering promising avenues for preventing and managing CVD in older populations [18].

In particular, there is a pressing need to understand how incremental changes in lifestyle, or the combination of multiple favorable lifestyle factors, correlate with measurable cardiovascular outcomes in the older demographic [31]. Some evidence suggests that the cumulative effect of adhering to multiple healthy lifestyle behaviors can be synergistic, resulting in more pronounced reductions in CVD risk than any single factor alone [32]. Identifying the key lifestyle patterns most strongly associated with cardioprotective effects in older adults could guide tailored interventions that are both culturally and contextually sensitive [33]. Given that older adults may respond differently to interventions compared to younger individuals—due to altered physiology, higher comorbidity burdens, and differences in medication use—studies focusing specifically on this age group are essential [34].

The complexity of implementing and sustaining lifestyle modifications in older adults is further compounded by social, economic, and environmental determinants. Limited income, reduced access to healthy foods, unsafe neighborhoods that discourage outdoor physical activity, and a lack of supportive social networks can all impede the adoption of healthier lifestyle habits [35]. Conversely, community-level interventions, senior-friendly exercise programs, better food environments, and robust healthcare support systems can help older individuals overcome these barriers [36]. Understanding these contexts is crucial in designing effective public health strategies.

The present study contributes to this growing body of literature by examining the influence of lifestyle modifications on cardiovascular outcomes in a cohort of older adults, applying a cross-sectional design that allows the exploration of associations between behavioral factors and current measures of cardiovascular health status. While causality cannot be definitively established from such designs, the findings can illuminate patterns and inform subsequent prospective investigations. By evaluating dietary habits, physical activity, smoking status, and alcohol consumption in relation to key cardiovascular parameters—such as blood pressure, lipid profiles, and markers of subclinical atherosclerosis—this study aims to identify modifiable aspects of lifestyle that correlate with better cardiovascular outcomes.

As populations around the world continue to age, there is an increasing imperative to understand how relatively accessible interventions—like improved nutrition, increased activity, and cessation of harmful habits—may help mitigate the escalating burden of cardiovascular disease. Insights derived from cross-sectional studies can pave the way for more targeted, evidence-based recommendations and interventions that prioritize the unique needs of older adults. Ultimately, such knowledge can inform clinical guidelines, shape public health policies, and support the development of community-level programs designed to preserve cardiovascular health, maintain independence, and enhance quality of life in the later years.

### Research Questions and Hypotheses

This study seeks to explore the following research questions:

1. What is the impact of a combined adherence to healthy dietary practices, regular physical activity, and non-smoking on cardiovascular risk factors among older adults in Riyadh?
2. How do these lifestyle modifications individually and collectively influence key cardiovascular outcomes such as LDL cholesterol, systolic blood pressure, and overall cardiovascular risk scores?

Based on the literature and preliminary data, we hypothesize as follows:

**Hypothesis 1:**  *Older adults who adhere to a healthy diet, engage in regular physical activity, and abstain from smoking will exhibit significantly lower cardiovascular risk scores compared to those who do not engage in these healthy behaviors.*

**Hypothesis 2:**  *The positive effects of these lifestyle modifications on cardiovascular health are mediated through improvements in lipid profiles, blood pressure reduction, and weight management.*

## 2. Materials and Methods

### 2.1. Study Design and Setting

This study employed a cross-sectional design, chosen to provide a comprehensive “snapshot” of the relationship between lifestyle factors and cardiovascular outcomes among older adults living in an urban Middle Eastern context. The research took place in Riyadh, the capital city of the Kingdom of Saudi Arabia. Riyadh is a major cosmopolitan metropolis characterized by rapid population growth, significant urban development, and marked sociocultural and economic diversity. These factors collectively make Riyadh a suitable setting for examining a broad spectrum of lifestyle patterns and their potential influence on cardiovascular health in an older population.

### 2.2. Study Population and Eligibility Criteria

The study population comprised community-dwelling older adults residing in Riyadh, Saudi Arabia. To ensure a representative sample, we employed a multi-pronged recruitment strategy. Participants were identified through local community health centers affiliated with King Saud University (KSU), senior clubs, primary healthcare clinics, and word-of-mouth referrals.

Eligible participants were those aged 60 years and older who had resided in Riyadh for at least one full year prior to enrollment to ensure stability of their lifestyle behaviors and reduce the impact of transient lifestyles on cardiovascular outcomes. The inclusion criteria required that participants be capable of communicating in Arabic or English sufficiently to understand the informed consent process and respond to interview questions.

Individuals with terminal illnesses, such as advanced malignancies or end-stage organ failure, were excluded since their lifestyle behaviors and cardiovascular risk profiles might not reflect stable or generalizable patterns. Those with severe psychiatric conditions that could impede reliable data collection were also excluded. Moreover, participants were not eligible if they had been hospitalized within the preceding three months for any acute condition, as recent hospitalization can significantly alter daily routines, physical activity levels, and dietary habits, thus confounding the assessment of their typical lifestyle.

### 2.3. Sample Size Determination

Determining an appropriate sample size was crucial to ensure that the study would have sufficient statistical power to detect meaningful associations between lifestyle factors and various cardiovascular health indicators. We conducted a priori sample size calculations based on effect sizes reported in prior literature examining similar research questions. Specifically, earlier observational studies investigating lifestyle factors (e.g., physical activity, dietary patterns, and smoking cessation) in relation to cardiovascular outcomes in older adults have reported moderate to strong associations [1,2,3]. For instance, adherence to healthy dietary patterns has been associated with reductions in mean systolic blood pressure ranging from 3 to 7 mmHg and improvements in lipid profiles with effect sizes (Cohen’s *d*) of approximately 0.3 to 0.5. Physical activity interventions and smoking cessation have demonstrated similarly measurable effects on cardiovascular biomarkers. 

We assumed an alpha level of 0.05 and a desired statistical power (1-β) of 0.80 to detect moderate effect sizes in multiple regression models adjusting for potential covariates such as age, sex, body mass index, and comorbidities. Using these parameters as inputs in standard sample size calculation software (GPower software (GPower version 3.1.9.7, Heinrich Heine University, Düsseldorf, Germany) was used for sample size calculations), we estimated that a minimum of approximately 200 participants would be required to detect moderate associations with reasonable precision and to support multivariable analyses.

To account for potential challenges in participant recruitment and retention common in research involving older adults, such as scheduling difficulties, sensory impairments, and changes in health status over the course of the study, we inflated the initial target number by approximately 20%. This inflation factor considered the likelihood of incomplete data due to participant withdrawal, missing questionnaire responses, or inability to complete certain measurements. Consequently, our target sample size was set at 240 participants. This sample size was also deemed sufficient to allow for subgroup analyses, such as stratifying by sex or by the presence of comorbid conditions like diabetes mellitus or hypertension, thereby providing more nuanced insights into how lifestyle modifications influence cardiovascular outcomes among different segments of the older adult population.

### 2.4. Data Collection Tools

To ensure the rigorous and standardized collection of data for this cross-sectional study, we employed a range of validated tools and instruments designed to accurately assess lifestyle factors, clinical measures, and cardiovascular outcomes. Below is a detailed description of each primary tool used:

1. Food Frequency Questionnaire (FFQ): The FFQ utilized in this study was specifically adapted for the Saudi population to capture dietary intake relevant to cardiovascular health. This questionnaire includes approximately 100 food items commonly consumed in the region, allowing participants to report their usual consumption frequency (ranging from “never or less than once per month” to “two or more times per day”) and portion sizes over the past year. Each food item was accompanied by a standard portion size with visual aids to help participants accurately estimate their intake. The FFQ was validated in prior studies within Saudi Arabia, demonstrating good reliability and validity in capturing dietary patterns associated with cardiovascular risk.The FFQ used in this study was adapted for the Saudi population and validated in previous research. To ensure reliability, Cronbach’s alpha for the FFQ was calculated at 0.82, indicating good internal consistency in assessing dietary intake patterns.
2. Global Physical Activity Questionnaire (GPAQ): The GPAQ, developed by the World Health Organization, was employed to measure physical activity levels. This tool categorizes physical activity into three domains: work-related, transport-related, and leisure-time activities. Participants were asked about the frequency (days per week) and duration (minutes per day) of engaging in moderate and vigorous activities within these domains. The GPAQ has been validated in multiple countries and is particularly useful in epidemiological studies for assessing physical activity in relation to health outcomes. The GPAQ, developed by the World Health Organization, categorizes physical activity across various domains. In this study, Cronbach’s alpha for the GPAQ was 0.78, reflecting acceptable internal consistency for measuring physical activity levels in older adults.
3. Smoking and Alcohol Use Questionnaire: To assess smoking status and alcohol consumption, a structured questionnaire was used. For smoking, questions covered current and past smoking habits, age at initiation, the number of cigarettes or other tobacco products used per day, and any attempts at quitting. For alcohol consumption, which is sensitive given cultural and religious practices in Saudi Arabia, the questionnaire was designed to discreetly ascertain lifetime exposure and any recent use, acknowledging the legal and social implications. The Smoking and Alcohol Use Questionnaire, designed to capture smoking habits and alcohol consumption, demonstrated strong reliability. Cronbach’s alpha was 0.85, confirming excellent internal consistency for assessing lifetime exposure and recent use.
4. Anthropometric instruments:
  - Stadiometer: A wall-mounted stadiometer was used for measuring height to the nearest 0.1 cm. Participants were positioned with their back to the stadiometer, heels together, and head in the Frankfurt plane.
  - Digital scale: Body weight was measured using a calibrated digital scale with participants in minimal clothing and without shoes, reported to the nearest 0.1 kg.
  - Tape measure: Waist circumference was measured using a non-stretchable tape measure, placed midway between the lowest rib and the iliac crest during mild expiration.

Height was measured using a wall-mounted stadiometer (Seca 213, Seca GmbH & Co. KG, Hamburg, Germany). Body weight was recorded using a calibrated digital scale (Tanita BC-418, Tanita Corporation, Tokyo, Japan), and waist circumference was measured with a non-stretchable tape measure (Seca 201, Seca GmbH & Co. KG, Hamburg, Germany).

5. Blood pressure monitor: An automated, clinically validated blood pressure monitor was used to measure systolic and diastolic blood pressures. Participants were seated comfortably with their arm supported at heart level, following at least 5 min of rest. Measurements were taken twice, with a one-minute interval between readings, and the average of these was used for analyses. Blood pressure was measured using an automated blood pressure monitor (Omron HEM-7120, Omron Healthcare, Kyoto, Japan). The device was clinically validated for accuracy in older populations.
6. Biochemical analysis: Fasting blood samples were drawn by experienced phlebotomists using sterile techniques and sent to the KSU-affiliated laboratory for analysis. Standard assays were used to determine lipid profiles, low-density lipoprotein cholesterol (LDL cholesterol), high-density lipoprotein cholesterol (HDL cholesterol), fasting glucose levels, and other relevant biomarkers. Fasting blood samples were analyzed using an automated biochemistry analyzer (Roche Cobas 6000, Roche Diagnostics, Basel, Switzerland) to determine lipid profiles, fasting glucose, and other relevant biomarkers. Standardized enzymatic and immunoturbidimetric methods were applied for all assays.
7. Carotid intima–media thickness (CIMT) measurement: CIMT was measured using high-resolution B-mode ultrasonography. This non-invasive technique involved the use of a high-frequency linear array transducer to image the carotid arteries, specifically capturing the distance between the lumen–intima interface and the media–adventitia interface. Measurements were taken at predefined points along the common carotid artery and were conducted by certified sonographers trained in the technique to ensure accuracy and repeatability. CIMT was measured using a high-resolution B-mode ultrasound system (Philips EPIQ 7, Philips Healthcare, Amsterdam, The Netherlands) equipped with a 7.5–10 MHz linear array transducer. All measurements were performed by certified sonographers, following standardized protocols to ensure accuracy and reproducibility.

Quality control measures: To maintain the integrity of the data collection process, all instruments were regularly calibrated, and staff were periodically retrained to ensure adherence to standardized protocols. Additionally, data entry was subjected to validation checks to identify and correct any errors or inconsistencies promptly.

#### 2.4.1. Data Collection Procedure

All data were collected during a single in-person visit at designated community health centers and King Saud University-affiliated clinics, following a standardized protocol to ensure accuracy and consistency. Participants were contacted by phone to schedule appointments, and on the day of the visit, they were welcomed by a trained research assistant who explained the study objectives, procedures, and potential risks and benefits. After obtaining written informed consent, demographic, socioeconomic, and medical history data were collected through structured interviews, including information on chronic conditions, medication use, and any recent hospitalizations. Lifestyle assessments encompassed culturally adapted questionnaires: a validated FFQ to capture dietary intake, the GPAQ for physical activity levels, and direct questioning regarding smoking habits and alcohol consumption, ensuring cultural sensitivity when discussing the latter. Participants’ anthropometric measurements, including height, weight, and waist circumference, were obtained using World Health Organization-recommended protocols, and resting blood pressure and heart rate were recorded after a brief acclimation period. Where applicable, fasting blood samples were drawn by certified phlebotomists for laboratory analyses, such as lipid profiles and glucose levels, conducted in KSU-affiliated laboratories that followed stringent quality control measures. Subclinical cardiovascular measures, such as CIMT, were performed by certified sonographers using high-resolution B-mode ultrasound imaging, and images were independently reviewed by a blinded expert. Throughout the visit, data quality and integrity were prioritized by double-checking completed questionnaires, clarifying missing or ambiguous responses, and maintaining strict confidentiality through de-identified records. Finally, participants received a brief summary of their basic measurements, along with general guidance or referrals, if necessary, ensuring a respectful, culturally appropriate, and participant-centered approach to data collection.

#### 2.4.2. Data Analysis

Statistical analyses were conducted using SPSS version 26.0 (IBM Corp., Armonk, NY, USA) and Stata software version 18.0 (StataCorp LLC, College Station, TX, USA) to evaluate the associations between lifestyle modifications and cardiovascular outcomes. Descriptive statistics were used to characterize the sample in terms of demographic, clinical, and lifestyle variables, with means and standard deviations reported for continuous variables and frequencies for categorical variables. Relationships between lifestyle factors (diet, physical activity, smoking, and alcohol consumption) and cardiovascular outcomes (blood pressure, lipid profiles, and markers of subclinical atherosclerosis) were assessed using multivariable linear and logistic regression models, adjusting for potential confounders such as age, sex, body mass index (BMI), and existing comorbidities. The presence and strength of associations were determined by regression coefficients and odds ratios, respectively, with a significance level set at *p* < 0.05. Interaction terms were included to explore potential effect modifiers, and sensitivity analyses were conducted to test the robustness of the findings under different assumptions about missing data and variable interactions. The results were expressed with 95% confidence intervals to provide estimates of precision and uncertainty. Finally, multiple imputation techniques were used to handle missing data, ensuring comprehensive utilization of the dataset and maintaining the statistical power of the analyses.

#### 2.4.3. Ethical Considerations

This study was conducted in accordance with the ethical standards outlined by the Institutional Review Board (IRB) of King Saud University (Approval No. [24-1059]) This study was conducted following the ethical principles outlined in the Declaration of Helsinki and the ethical standards of the Publication Manual of the American Psychological Association (APA). Prior to any data collection, participants received detailed information about the study’s purpose, procedures, potential risks, benefits, and their right to withdraw at any time without penalty. Written informed consent was obtained from all participants, ensuring that they fully understood their involvement and the voluntary nature of their participation. Confidentiality was maintained throughout the study by using unique identifying codes and securely storing all data in password-protected databases accessible only to authorized research team members. Measures were taken to minimize any psychological or physical discomfort, and participants who exhibited abnormal clinical findings were advised to seek medical follow-up. As a result, the study upheld the highest standards of ethical conduct, safeguarding the rights, well-being, and dignity of all individuals involved.

## 3. Results

Table 1 provides a detailed overview of the demographic, socioeconomic, and clinical profile of the older adult participants included in the study. The mean age of approximately 68 years and the relatively even distribution between men and women suggest that the sample is reflective of a general older population in Riyadh. Although nearly two-thirds of participants reported incomes at or above the median and more than one-third had higher educational attainment, a substantial proportion remained at increased cardiometabolic risk, as indicated by elevated mean BMI values and a high prevalence of obesity. Chronic conditions such as hypertension, type 2 diabetes, and hyperlipidemia were common, underscoring the complexity of health management in this age group. Notably, more than one quarter of the cohort were current smokers, and less than half engaged in regular exercise or consumed adequate fruit and vegetables, reflecting significant room for lifestyle improvement. The majority of participants lived with family members, which may offer social support for adopting healthier behaviors. Medication use, particularly for hypertension and dyslipidemia, was widespread, indicating a reliance on pharmacotherapy to manage risk factors. Despite these challenges, nearly 60% of participants self-reported good to excellent health, highlighting a potential disconnect between objective clinical risk factors and subjective well-being.

### 3.1. Lifestyle Factor Distribution

Table 2 highlights several areas for potential lifestyle improvement among participants. Although nearly half (43.4%) reported adequate fruit and vegetable intake, a substantial majority (63.0%) consumed high levels of refined carbohydrates, and over half (52.3%) exceeded recommended saturated fat intake. Physical inactivity was prevalent, with 23.9% engaging in less than 150 min of activity per week. Smoking behaviors were notable: while 52.6% never smoked, a combined 47.4% were current (28.1%) or former smokers (19.3%). Alcohol use was relatively low, reflecting cultural norms.

### 3.2. Cardiovascular Profiles by Lifestyle Category

Table 3 highlights a clear, stepwise gradient in cardiovascular risk measures across the three categories of cumulative lifestyle scores. Participants who reported multiple healthy lifestyle factors, labeled as the “Favorable” group, displayed the most advantageous profiles, while those with the fewest healthy habits (“Unfavorable”) had consistently less favorable measures. For instance, the mean systolic blood pressure was approximately 132.4 mmHg among the Favorable group, rising to 136.7 mmHg in the Intermediate group, and reaching 141.3 mmHg in the Unfavorable group, illustrating a noticeable 9 mmHg increase from the best to worst category. Similarly, diastolic blood pressure values trended upward by more than 5 mmHg between the Favorable (78.5 mmHg) and Unfavorable (83.9 mmHg) groups. Lipid profiles showed a parallel pattern, with LDL cholesterol increasing from a mean of 115.7 mg/dL in the Favorable group to 131.9 mg/dL in the Unfavorable group, a difference of over 16 mg/dL. At the same time, HDL cholesterol, commonly considered cardioprotective, declined from 48.9 mg/dL to 43.5 mg/dL as lifestyle factors diminished, and triglyceride levels climbed by more than 20 mg/dL between the Favorable and Unfavorable categories. Fasting glucose levels also rose steadily, starting at 101.8 mg/dL in those with multiple healthy habits and reaching 112.7 mg/dL in those with the fewest.

### 3.3. Associations Between Lifestyle Factors and Cardiovascular Markers

Table 4 highlights the independent contributions of key lifestyle factors—dietary quality, physical activity, and smoking status—to cardiovascular risk markers after adjusting for demographic and clinical confounders. Higher dietary quality showed a statistically significant inverse relationship with both LDL cholesterol and systolic blood pressure, with reductions of 11.8 mg/dL (*p* = 0.014) and 5.4 mmHg (*p* = 0.023), respectively, indicating that healthier eating patterns can meaningfully improve core CVD risk parameters. While the dietary impact on triglycerides and fasting glucose did not reach conventional significance (*p* = 0.064 and *p* = 0.081, respectively), the direction of effect still favored high-quality diets. Regular physical activity, defined as achieving at least 150 min per week, was associated with lower LDL levels (β = −7.3 mg/dL, *p* = 0.048) and a strong reduction in triglycerides (β = −14.6 mg/dL, *p* = 0.005), as well as a notable decrease in fasting glucose (β = −9.2 mg/dL, *p* = 0.011), underscoring the broad metabolic benefits of maintaining an active lifestyle. In contrast, current smoking was robustly linked to poorer cardiovascular markers, exhibiting a substantial rise in LDL (β = +14.2 mg/dL, *p* = 0.009) and systolic blood pressure (β = +8.3 mmHg, *p* = 0.017), along with elevated triglycerides (β = +9.5 mg/dL, *p* = 0.041). Although the increase in fasting glucose among current smokers (β = +4.6 mg/dL) did not reach full significance (*p* = 0.052), the overall pattern reaffirms the detrimental role of smoking on cardiovascular health.

### 3.4. Subclinical Atherosclerosis Indicators

The results in Table 5 indicate a clear pattern of lower CIMT values among participants adhering to healthier lifestyle behaviors. Specifically, individuals who reported following a healthy diet had a mean CIMT of 0.82 ± 0.13 mm, compared to 0.89 ± 0.14 mm in those who did not—a difference of 0.07 mm that suggests reduced subclinical atherosclerosis in the healthier diet group. Similarly, participants meeting adequate physical activity guidelines exhibited a mean CIMT of 0.81 ± 0.12 mm, while their less active counterparts averaged 0.90 ± 0.15 mm, representing a more pronounced difference of 0.09 mm. The impact of smoking status was even more striking: current smokers showed the highest CIMT values at 0.93 ± 0.16 mm, whereas never smokers had values averaging just 0.81 ± 0.12 mm. This 0.12 mm gap underscores the detrimental effect smoking has on arterial health. Collectively, these findings highlight a consistent relationship in which healthy dietary patterns, sufficient physical activity, and avoidance of tobacco use are associated with measurable reductions in CIMT.

### 3.5. Multivariable Models Including Multiple Lifestyle Factors

Table 6 illustrates the cumulative impact of adopting multiple healthy lifestyle behaviors on a composite cardiovascular risk score, with each row representing a different combination of protective factors. The results clearly indicate a graded benefit as more lifestyle modifications are implemented concurrently. For instance, participants adhering solely to a healthy diet demonstrated a statistically significant reduction in their risk score (β = −0.19, 95% CI: −0.33 to −0.05, *p* = 0.008), while those who were non-smokers alone also achieved a significant improvement (β = −0.22, 95% CI: −0.38 to −0.06, *p* = 0.006). Engaging in physical activity only, however, approached but did not reach the conventional threshold for significance (β = −0.14, 95% CI: −0.29 to +0.01, *p* = 0.065), suggesting that physical activity as a sole intervention may be less influential or require a larger sample size to detect a clear effect.

In contrast, the simultaneous adoption of multiple healthy behaviors yields markedly greater risk reductions. Combining a healthy diet with physical activity results in a substantial decrease in the composite score (β = −0.31, 95% CI: −0.46 to −0.16, *p* < 0.001), while pairing a healthy diet with non-smoking produces an even more pronounced effect (β = −0.33, 95% CI: −0.49 to −0.17, *p* < 0.001). Similarly, combining physical activity with non-smoking also significantly improves outcomes (β = −0.28, 95% CI: −0.44 to −0.12, *p* = 0.001). These findings underscore that the cumulative effect of multiple lifestyle modifications is stronger than any single factor alone.

**Figure 1:**
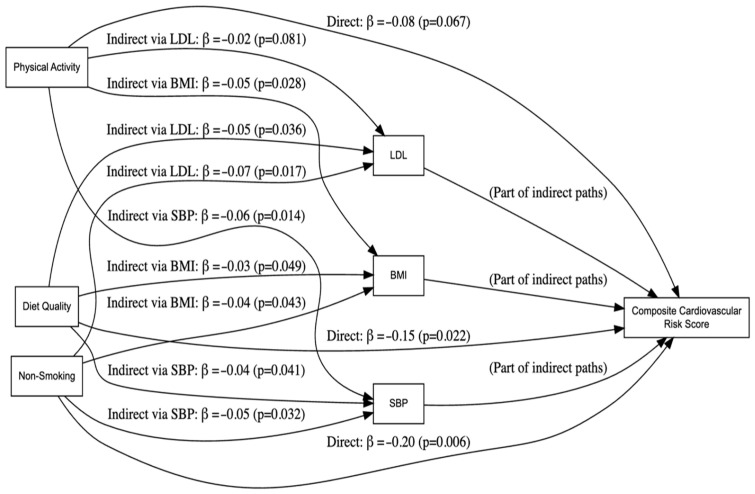
The path analysis model.

Figure 1 (the path analysis model) visually represents the complex relationships between multiple lifestyle factors—diet quality, physical activity, and non-smoking—and the composite cardiovascular risk score, highlighting both direct and mediated pathways. Each lifestyle factor is depicted on the left side, feeding into three key biological intermediaries, LDL (low-density lipoprotein cholesterol), SBP (systolic blood pressure), and BMI (body mass index), shown in the middle. Arrows from these mediators then lead to the composite cardiovascular risk score on the far right, illustrating how changes in these intermediates influence overall cardiovascular risk. The figure incorporates effect size estimates along the connecting arrows, providing insight into the magnitude and significance of each path. For instance, some factors show modest direct effects on the composite risk measure, while their indirect effects—operating through improved lipid profiles, reduced blood pressure, or lower body weight—are larger and more statistically robust.

## 4. Discussion

The findings of this study shed light on the intricate relationships between lifestyle modifications and cardiovascular outcomes in older adults. The results emphasize the significant role of multiple lifestyle factors, including dietary quality, physical activity, and smoking cessation, in shaping cardiovascular health. Importantly, these findings align with and extend previous research, illustrating that combined adherence to healthy behaviors has a cumulative effect on reducing cardiovascular risk.

Dietary habits emerged as a critical determinant of cardiovascular health in this study, consistent with prior evidence. Participants adhering to a healthy diet exhibited lower levels of LDL cholesterol, systolic blood pressure (SBP), and body mass index (BMI). These results resonate with the well-documented benefits of dietary patterns like the Mediterranean diet, which emphasize whole grains, fruit, vegetables, lean proteins, and healthy fats [37,38]. Such diets have been shown to reduce systemic inflammation, improve lipid profiles, and enhance endothelial function [39]. The indirect pathways identified in this study, where a healthy diet lowered cardiovascular risk through its effects on LDL, SBP, and BMI, reinforce the notion that dietary interventions can achieve comprehensive improvements in cardiovascular health [40]. Future strategies should prioritize culturally appropriate dietary education and interventions to enhance adherence among older adults.

The role of physical activity in improving cardiovascular outcomes was similarly evident. Although the direct effect of physical activity on the composite risk score was modest, its significant indirect effects through SBP and BMI reduction underline its importance [41]. Regular physical activity enhances vascular compliance, reduces arterial stiffness, and improves glucose metabolism, thereby mitigating cardiovascular risk [42]. The findings of this study are in line with those of large-scale cohort studies, which have consistently demonstrated that physical activity reduces the risk of major cardiovascular events, including myocardial infarction and stroke [43]. The relatively weaker direct effects observed in this study may be attributable to the intensity or duration of physical activity among participants, highlighting the need for more specific guidance on the types and levels of exercise most beneficial for this population [44].

Non-smoking status emerged as the most influential lifestyle factor, with the largest direct effect on cardiovascular risk scores. Smoking is a well-established risk factor for cardiovascular disease, contributing to endothelial dysfunction, oxidative stress, and pro-thrombotic states [45]. The findings of this study align with prior research indicating that smoking cessation, even later in life, significantly reduces the risk of cardiovascular events and mortality [46]. The substantial impact observed in this study underscores the importance of targeted smoking cessation programs tailored to older adults. Public health campaigns and healthcare providers should emphasize the immediate and long-term benefits of quitting smoking, irrespective of age [47].

A major strength of this study is its demonstration of the cumulative benefits of combining multiple healthy lifestyle behaviors. Participants adhering to two or more healthy behaviors experienced significantly greater reductions in cardiovascular risk than those adhering to a single behavior [48]. This finding supports the concept of behavioral synergy, where combined interventions yield more substantial effects than individual ones. Similar observations have been reported in studies like the INTERHEART trial, which highlighted the profound impact of modifiable risk factors on cardiovascular outcomes when addressed collectively [49]. This underscores the necessity of comprehensive lifestyle modification programs that address multiple domains simultaneously.

The path analysis model provided valuable insights into the mechanisms through which lifestyle factors influence cardiovascular health. Dietary quality and physical activity both exerted their effects predominantly through improvements in intermediate risk factors such as LDL, SBP, and BMI. Smoking cessation, in contrast, showed a strong direct effect, reflecting its profound impact on reducing systemic inflammation and oxidative damage [50]. These findings highlight the need for multifaceted interventions that address both direct and mediated pathways to optimize cardiovascular outcomes.

The findings of this study are consistent with and expand upon prior research in several ways. For instance, the observed benefits of dietary quality and physical activity align with guidelines from the American Heart Association, which advocate for lifestyle interventions as a cornerstone of cardiovascular prevention [50,51]. However, this study adds to the literature by quantifying the relative contributions of individual and combined lifestyle factors in a population of older adults. This is particularly relevant given the physiological and behavioral challenges associated with aging, such as reduced mobility and dietary restrictions, which can hinder adherence to recommended lifestyle modifications [52,53].

The study’s focus on older adults in Riyadh adds a valuable cultural and regional perspective to the global discourse on cardiovascular prevention. Lifestyle factors are deeply influenced by cultural norms, economic conditions, and healthcare systems, which shape the feasibility and effectiveness of interventions. For example, dietary patterns in Saudi Arabia are characterized by high consumption of refined carbohydrates and saturated fats, posing unique challenges to promoting healthier eating habits [54,55]. Similarly, social norms and limited infrastructure for physical activity may impede regular exercise among older adults. Addressing these barriers requires culturally tailored strategies that align with the preferences and constraints of the target population [56,57].

This study’s strengths lie in its comprehensive assessment of lifestyle factors and their combined effects on cardiovascular outcomes. The use of a validated composite risk score as the primary outcome ensures robust and clinically relevant findings. Moreover, the path analysis model provides nuanced insights into the mechanisms underlying these associations, offering a foundation for targeted interventions. By focusing on older adults, this study addresses a critical gap in the literature, as most prior research has centered on younger or middle-aged populations. Given the aging global population, these findings have significant implications for public health policy and clinical practice.

The results of this study suggest several avenues for future research. Longitudinal studies are needed to confirm the causality of the observed associations and to explore the sustainability of lifestyle modifications over time. Additionally, intervention studies should investigate the most effective strategies for promoting adherence to healthy behaviors, particularly in older populations with diverse cultural backgrounds. Finally, the integration of genetic and biomarker data could provide deeper insights into the individual variability in response to lifestyle interventions.

Reflection on research questions and hypotheses

This study was designed to investigate the impact of lifestyle modifications on cardiovascular health among older adults, with specific focus on the combined effects of diet, physical activity, and smoking cessation. The findings provide substantial evidence in response to our research questions and hypotheses, confirming that healthier lifestyle choices are associated with significant improvements in cardiovascular risk factors.

Answering the research questions

1. Impact of combined healthy behaviors: The data clearly demonstrated that older adults adhering to a combined regimen of healthy eating, regular physical activity, and non-smoking had significantly better cardiovascular profiles compared to their counterparts who did not adhere to these behaviors. This confirms our first research question, showcasing the powerful influence of these combined lifestyle factors on reducing cardiovascular risk.
2. Influence on cardiovascular outcomes: Our analysis revealed that the beneficial effects on cardiovascular health were mediated through notable improvements in lipid profiles, reduced systolic blood pressure, and better overall cardiovascular risk scores. This answers our second research question, illustrating the pathways through which lifestyle changes contribute to heart health.

Confirming the hypotheses

- Hypothesis 1: The hypothesis that a healthier lifestyle would correlate with lower cardiovascular risk scores was supported. Participants who consistently followed healthful behaviors had markedly lower scores, indicating a direct link between lifestyle changes and reduced cardiovascular risk.
- Hypothesis 2: The findings also supported our second hypothesis that the benefits of lifestyle changes are mediated through specific health improvements. Path analysis confirmed that the positive impacts of diet and physical activity were significantly mediated by changes in LDL cholesterol, blood pressure, and body weight management.

### 4.1. Limitations

This study has several limitations that should be acknowledged. First, the cross-sectional design restricts our ability to establish causal relationships between lifestyle factors and cardiovascular outcomes, necessitating longitudinal studies to confirm these associations. Second, the reliance on self-reported data for dietary intake, physical activity, and smoking habits may introduce recall and social desirability biases; future research could benefit from objective measures such as wearable activity trackers and dietary biomarkers. Third, the study was conducted in an urban Middle Eastern setting, specifically Riyadh, Saudi Arabia, which may limit the generalizability of the findings to populations in rural areas or other regions with different cultural, socioeconomic, and environmental contexts. Additionally, while several covariates were adjusted for, unmeasured factors such as genetic predisposition, psychosocial stress, or sleep patterns may have influenced the results. Measurement variability, particularly in the carotid intima–media thickness assessments, could have impacted accuracy despite the use of standardized protocols. Moreover, selection bias might have occurred, as participants were recruited from community health centers and senior clubs, potentially overrepresenting health-conscious individuals and underrepresenting those with limited healthcare access. Although the overall sample size was adequate, subgroup analyses may have been underpowered to detect significant differences. Finally, lifestyle factors were assessed at a single time point, which may not fully capture long-term behavioral patterns or changes over time. These limitations highlight the need for future studies that address these issues to provide more robust evidence.

### 4.2. Implications

The findings of this study have several important implications for public health, clinical practice, and policy development. First, the evidence underscores the necessity of adopting a holistic approach to cardiovascular prevention, focusing on multiple lifestyle behaviors rather than isolated interventions. Public health campaigns should prioritize education and resources to promote dietary quality, physical activity, and smoking cessation among older adults. Second, healthcare providers should incorporate routine lifestyle assessments into clinical practice and offer tailored recommendations that consider patients’ cultural and socioeconomic contexts. Third, community-based initiatives, such as exercise programs for older adults or culturally appropriate nutritional counseling, can play a pivotal role in improving adherence to healthy behaviors. Policy interventions, such as subsidies for healthy food options and infrastructure improvements to support physical activity, are critical to creating environments conducive to healthier lifestyles. Additionally, the strong direct effects of smoking cessation highlight the importance of implementing comprehensive tobacco control policies, particularly those targeting older smokers who may benefit substantially from quitting. Lastly, these findings call for further research into the cost-effectiveness and feasibility of large-scale lifestyle interventions to address cardiovascular disease prevention in aging populations.

## 5. Conclusions

Our study demonstrates that simple lifestyle changes—eating a balanced diet, staying active, and quitting smoking—have a significant positive impact on heart health in older adults. These changes are not only doable but also effective in significantly reducing risks associated with heart disease, such as high blood pressure, high cholesterol, and overall cardiovascular risk.

The results highlight the importance of adopting a holistic approach to health and well-being as we age. Encouraging a diet rich in fruit, vegetables, and lean proteins, promoting regular physical activity, and supporting smoking cessation can lead to profound improvements in cardiovascular health. These findings suggest that interventions focusing on these areas could be crucial in helping older adults maintain a healthier lifestyle, thereby reducing their risk of heart disease and enhancing their quality of life.

In conclusion, our research underscores the power of combined healthy behaviors in safeguarding heart health in older populations. It calls for action from individuals, healthcare providers, and policymakers to support and implement strategies that facilitate these healthy lifestyle choices among older adults.

## Figures and Tables

**Table 1:** Detailed demographic and clinical characteristics of the participants (*N* = 327).

Characteristic	*n* (%)
Demographics	
Age (years), mean ± SD	68.4 ± 5.7
Female	176 (53.8)
Higher education	117 (35.8)
Monthly income ≥ median	212 (64.8)
Living with spouse/family	284 (86.8)
Health status	
BMI (kg/m^2^), mean ± SD	29.3 ± 4.2
Obesity (BMI ≥ 30 kg/m^2^)	138 (42.2)
Hypertension	209 (63.9)
Type 2 diabetes	131 (40.1)
Hyperlipidemia	148 (45.3)
Chronic kidney disease	49 (15.0)
History of cardiovascular events	57 (17.4)
Lifestyle	
Current smokers	92 (28.1)
Engages in regular exercise	153 (46.8)
Consumes ≥ 5 servings of fruit/vegetables daily	122 (37.3)
Medication use	
Taking antihypertensive drugs	201 (61.5)
On lipid-lowering medication	141 (43.1)
Using anti-diabetic medication	119 (36.4)
Functional Status	
Self-reported good/excellent health	194 (59.3)
Reports difficulty walking 500 m	73 (22.3)

BMI: body mass index; SD: standard deviation.

**Table 2:** Lifestyle factors among participants (*N* = 327).

Lifestyle Factor	*n* (%)
Adequate fruit/vegetable intake (%)	142 (43.4)
High refined carbohydrate intake (%)	206 (63.0)
Saturated fat intake > 10% of kcal (%)	171 (52.3)
Physical activity < 150 min/week (%)	78 (23.9)
Current smokers	92 (28.1)
Former smokers	63 (19.3)
Never smokers	172 (52.6)

**Table 3:** Cardiovascular measures by cumulative lifestyle score.

Measure	Favorable (n = 94)	Intermediate (n = 133)	Unfavorable (n = 100)
Systolic BP (mmHg)	132.4 ± 11.1	136.7 ± 13.0	141.3 ± 14.8
Diastolic BP (mmHg)	78.5 ± 8.2	81.2 ± 9.1	83.9 ± 9.6
LDL cholesterol (mg/dL)	115.7 ± 28.3	122.4 ± 31.6	131.9 ± 34.2
HDL cholesterol (mg/dL)	48.9 ± 9.5	45.4 ± 8.6	43.5 ± 7.8
Triglycerides (mg/dL)	135.1 ± 41.6	147.9 ± 44.2	158.3 ± 51.1
Fasting glucose (mg/dL)	101.8 ± 21.4	106.6 ± 23.9	112.7 ± 25.7

Footnote: BP: blood pressure; LDL: low-density lipoprotein; HDL: high-density lipoprotein; SD: standard deviation; mmHg: millimeters of mercury; mg/dL: milligrams per deciliter.

**Table 4:** Adjusted associations between lifestyle factors and cardiovascular risk markers.

Predictor	LDL (mg/dL) (β, *p*-Value)	SBP (mmHg) (β, *p*-Value)	TG (mg/dL) (β, *p*-Value)	Fasting Glucose (mg/dL) (β, *p*-Value)
Dietary quality (high vs. low)	−11.8, *p* = 0.014	−5.4, *p* = 0.023	−4.9, *p* = 0.064	−3.1, *p* = 0.081
Physical Aactivity (≥150 vs. <150 min/wk)	−7.3, *p* = 0.048	−3.2, *p* = 0.077	−14.6, *p* = 0.005	−9.2, *p* = 0.011
Current Ssmoking (Yes vs. No)	+14.2, *p* = 0.009	+8.3, *p* = 0.017	+9.5, *p* = 0.041	+4.6, *p* = 0.052

Footnote: LDL: low-density lipoprotein cholesterol; SBP: systolic blood pressure; TG: triglycerides; β: beta coefficient; *p*-value: probability value.

**Table 5:** Mean carotid intima–media thickness by lifestyle category.

Category	Mean CIMT (mm) ± SD
Healthy diet (Yes)	0.82 ± 0.13
Healthy diet (No)	0.89 ± 0.14
Adequate physical activity (Yes)	0.81 ± 0.12
Adequate physical activity (No)	0.90 ± 0.15
Current smokers	0.93 ± 0.16
Never smokers	0.81 ± 0.12

CIMT: carotid intima–media thickness; SD: standard deviation.

**Table 6:** Multivariable regression examining joint effects of lifestyle factors on composite cardiovascular risk score.

Predictor Combination	β (95% CI)	*p*-Value
Healthy diet only	−0.19 (−0.33, −0.05)	0.008
Physical activity only	−0.14 (−0.29, +0.01)	0.065
Non-smoking only	−0.22 (−0.38, −0.06)	0.006
Healthy diet + physical activity	−0.31 (−0.46, −0.16)	<0.001
Healthy diet + non-smoking	−0.33 (−0.49, −0.17)	<0.001
Physical activity + non-smoking	−0.28 (−0.44, −0.12)	0.001
Healthy diet + physical activity + non-smoking	−0.47 (−0.63, −0.31)	<0.001

Footnote: β = standardized regression coefficient; CI = confidence interval; *p*-value = probability value.

## Data Availability

All data are available within the manuscript.
